# *Porphyra yezoensis* Sauces Fermented With Lactic Acid Bacteria: Fermentation Properties, Flavor Profile, and Evaluation of Antioxidant Capacity *in vitro*

**DOI:** 10.3389/fnut.2021.810460

**Published:** 2022-01-13

**Authors:** Jie Yang, Tengqi Gao, Feng Ge, Hao Sun, Zihang Cui, Zhen Wei, Shujun Wang, Pau Loke Show, Yang Tao, Wenbin Wang

**Affiliations:** ^1^Jiangsu Key Laboratory of Marine Bioresources and Environment/Jiangsu Key Laboratory of Marine Biotechnology, Jiangsu Ocean University, Lianyungang, China; ^2^Jiangsu Key Laboratory of High-Tech Research and Development of Veterinary Biopharmaceuticals, Jiangsu Agri-Animal Husbandry Vocational College, Taizhou, China; ^3^Co-Innovation Center of Jiangsu Marine Bio-industry Technology, Jiangsu Ocean University, Lianyungang, China; ^4^Department of Chemical and Environmental Engineering, Faculty of Science and Engineering, University of Nottingham Malaysia, Semenyih, Malaysia; ^5^College of Food Science and Technology, Nanjing Agricultural University, Nanjing, China

**Keywords:** *Porphyra yezoensis* sauce, lactic acid bacteria, volatile components, GC-MS, antioxidation

## Abstract

The demand for roasted seaweed sandwich (*Porphyra yezoensis*) product has risen in recent years. The product slicing process has created a huge number of scraps that are not utilized effectively. Three lactic acid bacteria (LAB) strains were used to ferment *P. yezoensis* sauces in this study, including *Lactobacillus fermentum, Lactobacillus casei, Streptococcus thermophilus*, and the mixed strains (1:1:1, v/v). The fermentation characteristics, antioxidant capacity *in vitro*, sensory properties, and flavoring substances of fermented *P. yezoensis* sauces were analyzed. After 21 days of fermentation, all LAB strains grew well in the *P. yezoensis* sauces, with protease activity increased to 6.6, 9.24, 5.06, and 5.5 U/mL, respectively. Also, the flavors of *P. yezoensis* sauces fermented with *L. casei* and *L. fermentum* were satisfactory. On this premise, gas chromatography-mass spectrometry (GC-MS) was used to investigate the changes in gustatory compounds in *P. yezoensis* sauces fermented with *L. casei* and *L. fermentum*. In general, 42 and 41 volatile flavor chemicals were identified after the fermentation of *L. casei* and *L. fermentum*. Furthermore, the fermented *P. yezoensis* sauce possessed greater DPPH scavenging activity and ferric-reducing ability power than the unfermented *P. yezoensis*. Overall, the flavor and taste of *P. yezoensis* sauce fermented by *L. casei* was superior.

## Introduction

The East China Sea, South China Sea, Huanghai Sea, and other coastal regions produce *Porphyra yezoensis*. The protein level in dried *P. yezoensis* is 25–30%, while the carbohydrate amount is around 40% (1). It also contains vitamins like riboflavin and niacin (2). Moreover, *P. yezoensis* is rich in minerals that can assist youngsters and the elderly to absorb nutrition (3, 4).

Dietary *P. yezoensis* products are popular in Japan and Korea while Chinese consumers eat less *P. yezoensis* products. The local market of *P. yezoensis* products in China is underdeveloped with few product varieties and influential manufactors (5). One of the main reasons is that *P. yezoensis* processing are essential, whereas the products have a distinct flavor that some domestic consumers cannot tolerate. Currently, the primary domestic seaweed processing methods are drying and roasting. Thus, more processing methods of *P. yezoensis* are needed to boosting its economic value and increase the market of *P. yezoensis* products.

The slicing of roasted seaweed sandwich into small pieces generates a large number of wastes, which are rich in protein and maltose. The processing of sandwich seaweed filet to *Porphyra* sauce helps save waste. In addition, microbal fermentation, expecially lactic acid fermentation can preserve the nutritional value of *Porphyra* sauce, give it a unique taste and flavor, enrich its product form, and creat new opportunities for the *Porphyra* market. To our knowledge, the fermentation of *P. yezoensis* sauces has been poorly studied. Zhang et al. investigated the basic nutrient content of dried *Porphyra*, and fermented it with LAB and *Aspergillus oryzae* (6). Fan et al. utilized *A. oryzae* and *Rhodozyme* to co-ferment *Porphyra* and optimized the fermentation based on the protease activity and sensory index of *Porphyra* sauces (7).

LAB is a probiotic that may generate high quantities of amino acids in its fermentation *P. yezoensis* supernatant. It coud be a suitable species to ferment *Porphyra* sauces (8). This study chose *L. fermentum, L. casei*, and *S. thermophilus* to independently and co-ferment waste materials of roasted seaweed sandwich. The changes of fermentation characteristics and flavor profile were evaluated. Furthermore, the DPPH scavenging activity and ferric-reducing ability power (FRAP) of fermented *P. yezoensis* sauces were also investigated. The findings may help to produce probiotic fermented *P. yezoensis* sauces with excellent nutritional value and flavor.

## Materials and Methods

### Bacterial Strains and Culture Conditions

*S. thermophilus* FJAT-46738, *L. fermentum* FJAT-46744, and *L. casei* FJAT-7928 were obtained from the Fujian Academy of Agricultural Sciences, China. The single colony of the three LAB strains were innoculted to 10 mL MRS broth, cultivated for 36 h at 40°C except for *L. fermentum* (37°C). These strains were cultured with the AnaeroPack System C-32 (Mitsubishi Gas Chemical Company, Inc. Tokyo).

### Fermentation of *P. yezoensis* Sauce

The waste materials of roasted seaweed (*P. yezoensis*) sandwich was obtained from a local plant (Lianyungang Wende Food Co., Ltd. Lianyungang) in Lianyungang, Jiangsu, China. The seaweed sandwich scraps were crushed to a specific value (0.01 mm) by a beater. The aperture of the machine sieve hole should be kept at around 0.01 mm to promote the release of protein from the tissue (9). Ten grams of seaweed sandwich scrap powders and 15 mL water were mixed with a ratio (m/v) of 1:1.5 in 100 mL glass bottles with blue lid. The bottles were then sterilized at 121°C for 30 min in a autoclave. This process both softened the tissue of *P. yezoensis* and inactivated the residue bacteria. After cooling to room tempreature, the activated *L. fermentum, S. thermophilus, L. casei*, and their mixtures (1:1:1 v/v) were, respectively, added into the bottles at a ratio of 3% (v/v). The blue lid bottles were then tightened and statically fermented at 40°C. The viable bacteria level, pH, protease vitality, and sensory quality of the fermented products were measured at 0, 3, 7, 14, and 21 days.

### Determination of Viable Cell and pH

One gram of fermented *P. yezoensis* sauces were weighed and serially diluted with sterile 0.9% (w/v) NaCl solution. One hundred microlilter of each dilutions were, respectively, spread on MRS agar plates with two replicates and incubated in AnaeroPack System C-32 at 40°C for 36 h. The cell counts were expressed as colony numbers (Log 10 CFU per g). The *P. yezoensis* sauce without fermentation was used as control. An appropriate amount of fermented *P. yezoensis* sauces were sampled and the pH was measured by a digital pH meter (Instrument &amp; Electricity Scientific Instruments Company, Shanghai).

### Sensory Evaluations

Ten grams of each fermented *P. yezoensis* sauce were placed in plastic cups with cup and individually tasted by the sensory panelists. The sensory panel consisted of 7 persons (2 males and 5 females, aged 19–24) from School of Food Science and Engineering, Jiangsu Ocean University. All of the members were well-tranined before sensory evaluation. The sensory descriptors agreed by the team members include intensities (seaweed flavor, sauce flavor and no fishy flavor), preferences (aroma, sour flavor, bitter flavor and aftertaste), and overall quality. The intensity, preference, and overall quality scales ranged from 0 (weak) to 10 (strong), 0 (bad or dislike) to 10 (good or like), and 0 (dislike) to 10 (like), respectively (10).

About 10 g fermented *P. yezoensis* sauces at 25°C was randomly taken in the training and evaluation process. Water and white bread were provided to the assessors to wash the palate. The training and evaluation were organized in conformity to the International Organization for Standardization (ISO, 1993) and conducted in a sensory laboratory that complies with the American Society for Testing and Materials (ASTM) criteria.

### Determination of Protease Activity

The protease produced during the fermentation could effectively enhanced biological activity of the products (11). The formaldehyde method was used to determine the protease activity of *P. yezoensis* sauce (12). Ten grams of fermented *P. yezoensis* sauce in a 250 mL tapered container was added with 80 mL 55°C ddH_2_O, and incubated in water bath at 55°C for 3 h. The samples were then boiled for 1 min to inactivate the inherent enzyme. The sample was cooled to room temperature and the volume was adjusted to 100 mL with ddH_2_O, and filtered through filter paper. Ten milliliter of the filtrate in a 150 mL tapered bottle was added with 50 mL ddH_2_O, 4–5 drops of phenolphthalein indicator, and titrated with 0.1 mol/L NaOH solution until the solution just turned red. The volume of consumed NaOH was recorded as V1. The sample in the tapered bottle was added with 10 mL formaldehyde, titrated with 0.1 mol/L NaOH to dark red as the endpoint. The volume of consumed NaOH was recorded as V2. The protease viability is then calculated as (Eq. 1):


(1) $$\begin{array}{l} \text{Protease viability=}\{[(\text{V2-V1})\times \text{C}\times \text{0}.\text{044}]\text{/}\\ \;[\text{10}\times \text{10/100}]\}\times \text{100/1-W}n \end{array}$$

where V1 is the volume of NaOH consumed by titration before adding formaldehyde, mL; V2 is the volume of NaOH consumed by titration after adding formaldehyde, mL; C is the concentration of standard NaOH, mol/L; W is the moisture content of fermented *P. yezoensis* sauces, mL.

### GC-MS Analysis

Agilent 5977A series GC/MSD system (Agilent Technologies, CA) and Agilent HP-INNOWAX (30 m ×0.25 mm ×0.25 μm) were used to analyze the volatile compounds in the fermented *P. yezoensis* sauces. Two grams of sauce sample in a 20 mL headspace bottle was added with 3 mL of saturated sodium chloride solution (refrigerated at −20°C). The intake temperature was 250°C and the initial column temperature was 40°C. After incubation for 1 min, it was heated to 80°C at a rate of 4°C/min. This temperature was hold for 1 min, then heated to 160°C at a rate of 2°C/min, and finally heated to 220°C at a rate of 10°C/min. The temperature was then maintained for 10 min. Helium gas with high purity was used as the gas load and the flow rate was 1 mL/min. MS temperatures of the quaternary rod were 150°C. The ion source (230°C) was an electron-ion source with an electron energy of 70 eV and a scanning range of 35–350 *m/z*.

The semi-qualitative analysis was done with mass spectrometry in the computer spectral library (NIST/WILEY). The compound was identified when matched to a known volatile component with a score ≥80. The internal standard in this study was 2-octanol (concentration of 10 mg/L) (13, 14). The content of each volatile compound in the tested samples was calculated according to the following formula (Eq. 2).


(2) $$\begin{array}{r} C_{\text{X}}=M_{\text{X}}/M_{\;intensor}\times C_{\;intensor}\left(mg/kg\right) \end{array}$$

where CX is the concentration of volatile compounds to be measured, MX is the peak area of the measured volatile compound, M _intensor_ is the peak area of the internal standard, C _intensor_ is the concentration of the internal standard.

The data of samples were sorted and drawn with Origin 2021 to analyze the changes in volatile profile (15).

### Assay of DPPH Radical Scavenging and Ferric-Reducing Activity

Active ingredients of each *P. yezoensis* sauce (5 g) was extracted with 50 mL ddH_2_O. Ultrasound (Wuxi Gangzheng Technology Co., Ltd. Wuxi) was utilized to disrupt the cell wall and assist the extraction. The ultrasound power was 880 W the ultrasonic duration was 30 min, and the ultrasonic temperature was 50°C. The sample was then centrifuged at 8,000 g for 15 min, and the supernatant was collected (16, 17). *P. yezoensis* sauce extract (1 mL) was mixed with 1 mL of DPPH solution (0.1 mM), and incubated at 37°C in the dark for 30 min prior to measuring the absorbance at 517 nm (17). The scavenging activity of the DPPH radical was calculated as follows (Eq. 3).


(3) $$\begin{array}{l} \text{DPPH radical scavenging activity}(\%)\text{ =}\\ \text{ 1-}[({\text{A}}_{\text{ sample}}\text{- }{\text{A}}_{\text{ control}}){\text{/A}}_{\text{blank}}]\times \text{100} \end{array}$$

A _control_ was made by using absolute ethanol instead of DPPH ethanolic solution, and A _blank_ was prepared by replacing the polysaccharide sample solution with ddH_2_O.

The Ferric-Reducing activity of fermented *P. yezoensis* sauces were determined by the FRAP assay with an commercial kits (Jiancheng Bioengineering Institute, Nanjing, China). The absorbance was recorded at 593 nm.

### Statistical Analysis

All of the experiments were repeated triplicate. Data were expressed as the mean ± standard deviation. The data were analyzed by Excel, Origin 2021, and SPSS 20.0 software. Significance was defined at *p* < 0.05.

## Results

### Growth of LAB and pH Changes

**Figure 1:**
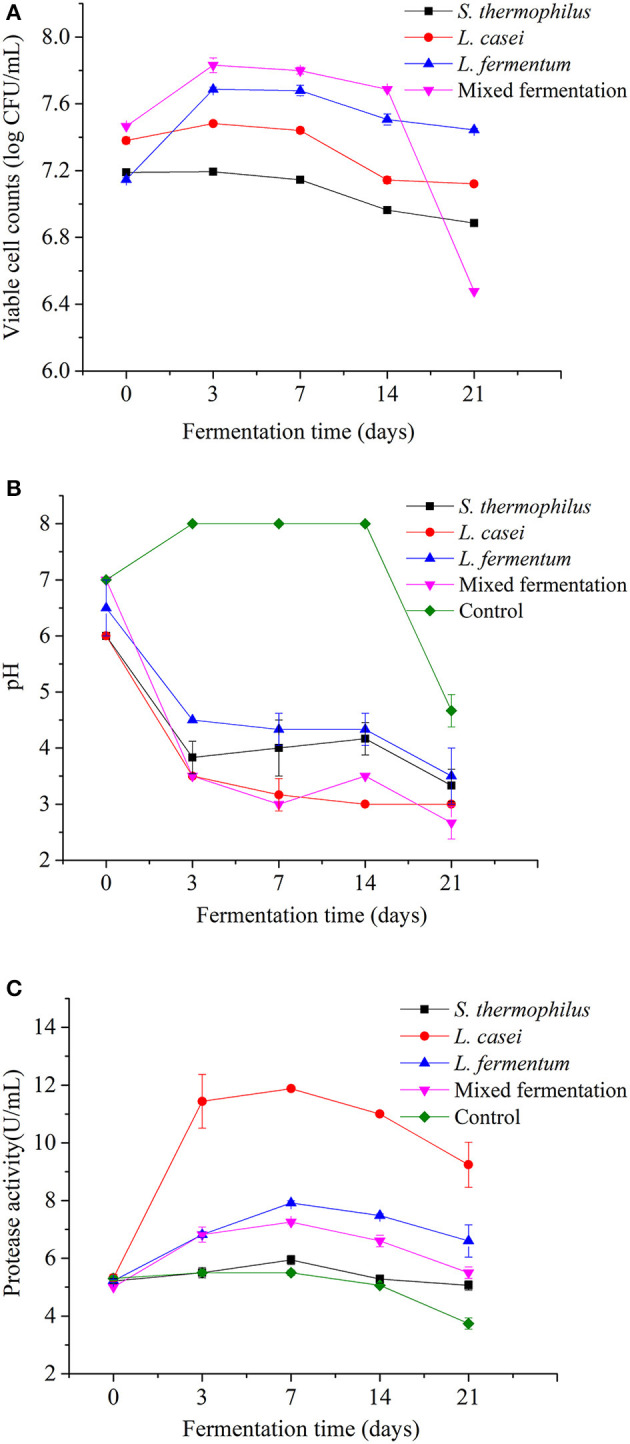
Evolutions of viable cell counts **(A)**, pH value **(B)** and protease activity **(C)** of *Porphyra yezoensis* sauces during 21 days of LAB strains fermentation.

The changes of viable cell counts and pH value of *P. yezoensis* sauces fermented with *S. thermophilus, L. casei, L. fermentum*, and the mixed strains during 21 days of fermentation are shown in Figure 1. All the three LAB strains were able to grow in the rehydrated roasted seaweed sandwich scraps. The initial viable bacteria counts of *L. casei, L. fermentum, S. thermophilus* and the mixed fermentation group were 7.38 ± 0.02, 7.15 ± 0.01, 7.19 ± 0.01 and 7.47 ± 0.03 log CFU/mL, respectively. After 21 days of fermentation, the viable cell counts in *P. yezoensis* sauce fermented with *L. casei, L. fermentum, S. thermophilus* and the mixed strains were 7.1 ± 0.01, 7.4 ± 0.01, 6.9 ± 0.015 and 6.5 ± 0.01 CFU/mL, indicating that the *L. casei* and *L. fermentum* strains were able to adapt to the fermentation environment. The number of viable bacteria of *P. yezoensis* sauce fermented with the mixed strains was considerably low at the end of fermentation. This was possibly due to the low pH (2.7 ± 0.29), which was one of the important factors affecting the number of viable bacteria during lactic acid fermentation (18). After 3 days of fermentation, the viable cell count frist rose and then decreased in the fermented *P. yezoensis* sauces. In contrast, no viable bacteria were found in the unfermented *P. yezoensis* sauce that was previously sterilized.

The pH value of the *P. yezoensis* sauce decreased during fermentation with all the LAB strains (Figure 1B). The pH values of the three strains have generally decreased. From 0 to Day 3, the pH value of fermented *P. yezoensis* sauces decreased the fastest. The pH value of each strain steadily dropped over a period of 3–21 days. When compared to the other two bacteria, *L. casei* has always had the lowest pH value. A low pH would efficiently inhibit the multiplication of pathogenic bacteria in samples and extend the shelf life (19).

### Determination of Protease Activity

Microbial fermentation might enhance the protease activity of the product (20). Figure 1C revealed that the protease activity of *P. yezoensis* sauces was initially around 5.20 U/mL and increased during all the fermentation processes. The protease activity increased to the peak at Day 7 and then decreased in the following fermentation period. Among all the groups, the protease activity of *P. yezoensis* sauces fermented by *L. casei* were the greatest (11.88 U/mL at day 7) at the same stage, which was partly consistent with the low pH value. After 21 days of fermentation, the protease activities of *P. yezoensis* sauces fermented by *L. fermentum, L. casei, S. thermophilus*, and the mixed strains were increased to 6.60, 9.24, 5.06, and 5.50 U/mL, respectively. Furthermore, the protease activity of all fermented *P. yezoensis* sauces were significantly increased (*p* < 0.05) than the control.

### Sensory Evaluation

**Figure 2:**
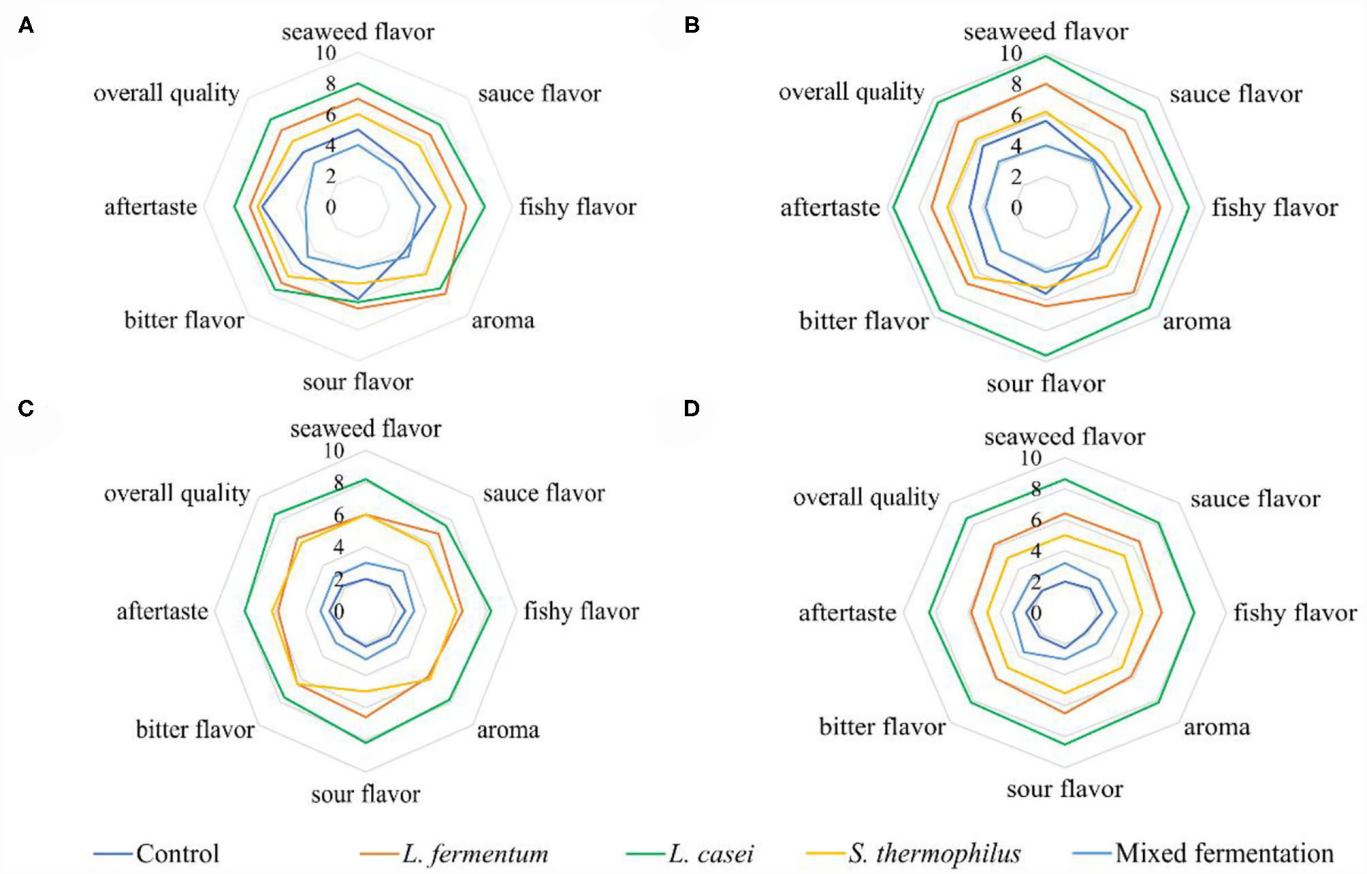
Radiation map of sensory profile for fermented *P. yezoensis* sauces. **(A)** 3 days of fermentation, **(B)** 7 days of fermentation, **(C)** 14 days of fermentation, **(D)** 21 days of fermentation.

The intensities of all the sensory attributes of fermented *P. yezoensis* sauces showed an increasing trend within the first week and then decreased gradually (Figure 2). The sensory quality of *P. yezoensis* sauces fermented by *L. casei* was the best among all the samples at the same fermentation period, followed by *L. fermentum, S. thermophilus* and the mixed strains. The fishy flavor, bitter flavor, and sour tastes in all samples got intensified with time, and the scent of seaweed and sauce flavor were more apparent in the *L. casei* fermented *P. yezoensis* sauce. Moreover, the sensory properties of the *P. yezoensis* sauce fermented by *L. fermentum* were relatively stable, and no significant changes in the flavors werwe observed. The aroma value increased the maximum at Day 7, and then declined significantly. Besides, the sensory quality of *P. yezoensis* sauces fermented by *S. thermophilus* were relatively low, among which the bitter and fishy smells were stonger than the aroma and sauce flavors. Taking all the sensory results into account, *L. casei* was more suitable for the *P. yezoensis* sauce fermentation.

### Analysis of Volatile Composition

**Figure 3:**
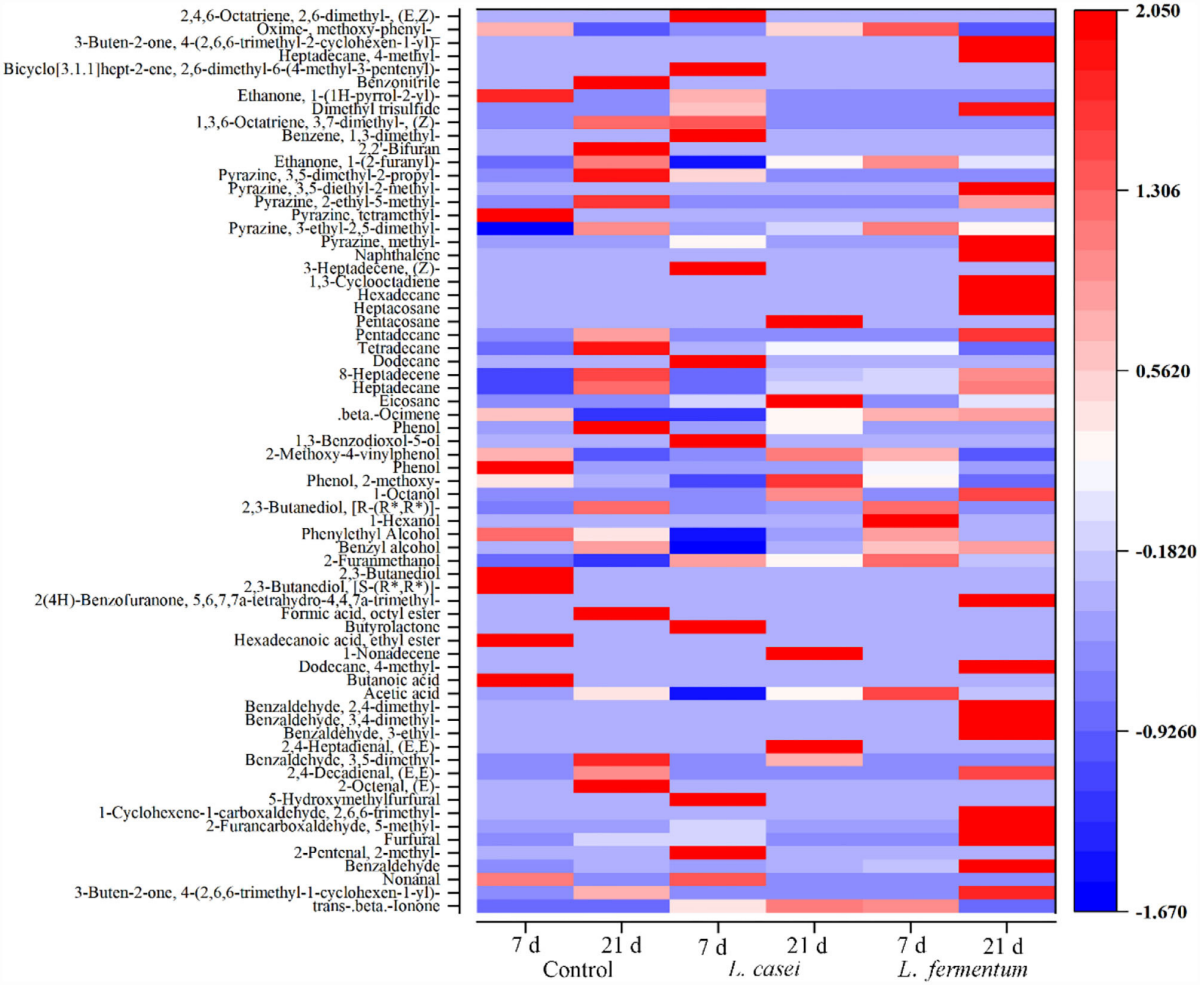
Heat map of volatile substances of *P. yezoensis* sauces.

According to the above results, *L. casei* and *L. fermentum* strains were preferred for fermenting *P. yezoensis* sauce. Thus, GC-MS was employed to investigate flavor alterations in *P. yezoensis* sauce fermented by *L. casei* and *L. fermentum*. Figure 3 shows that during fermentation at 7 and 21 day, a total of 67 volatile compounds were detected from the *P. yezoensis* sauces. Specifically, 35, 41, and 42 volatile compounds were identified in the control group, *L. fermentum* group and *L. casei* group during fermentation, respectively. These volatile compounds mainly included aldehydes, alcohols, hydrocarbons, acids, phenols, ketones, esters, pyrazines, furans and benzodiazepines and others (Supplementary Table 1). At a given fermentation, the volatile profile changed with time due to the catabolic response of flavor precursor material (21). Alcohols presented the highest amount in all the 6 samples, accounting for 65.62%. Aldehydes and hydrocarbons accounted for 46.45 and 27.29% of the total volatile compounds, respectively. Alcohols and esters were mainly produced in the middle stage of fermentation, while hydrocarbons were produced in the later stage (22).

### Assay of DPPH Radical Scavenging and Ferric-Reducing Activity

**Figure 4:**
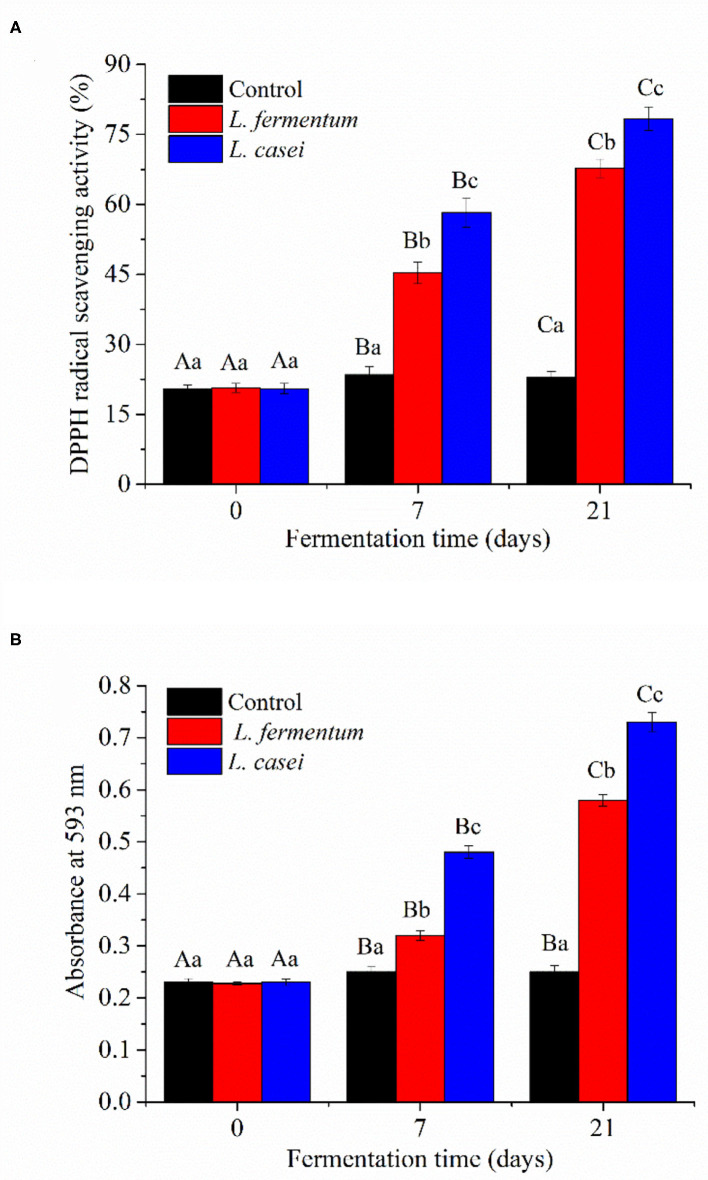
DPPH radical scavenging activity **(A)** and FRAP **(B)** of *P. yezoensis* sauces during fermentation. Values with different letters within each samples indicated a significant difference in content (*p* < 0.05).

Polysaccharides, polyphenols, proteins, and porphyrins were the physiologically active compounds found in *P. yezoensis*, with anticancer, antioxidant, hypolipidemic, and anti-inflammatory properties (23, 24). Fermentation with LAB can increase the biological activity of food by bio-transforming active molecules (25). The antioxidant capacities of *P. yezoensis* sauces extract before and after fermentation with *L. casei* and *L. fermentum* are shown in Figure 4. The free radical scavenging capability and ferric reducing antioxidant capacity of unfermented *P. yezoensis* sauce were only 20.4% and 0.23, respectively. The antioxidant activity of all the tested extract from fermentated *P. yezoensis* sauces steadly increased during the 21 days of fermentation. For the *P. yezoensis* sauce fermented by *L. casei* for 21 days, the free radical scavenging capability and ferric reducing antioxidant capacity increased to 78% and 0.73, respectively. Similarly, *L. fermentum* fermented samples increased to 67.8% and 0.58, respectively.

## Discussion

As more people become aware of the health benefits of laver, the laver industrial development has become more diverse and extended (26). The waste from the manufacturing of roasted seaweed sandwich were used to prepare the fermented *P. yezoensis* sauce. The obtained results revealed that *L. casei* was capable to grow in the the *P. yezoensis* sauce. The resulting seaweed sauce was featured by high enzyme vitality, and pronounced taste and flavor. In addition, compared with the control group and *L. fermentum* fermentation, *L. casei* fermentation improved the antioxidant activity of the sauce significantly, which was in line with the finding reported by Zubaidah et al., who fermented cabbage by *L. casei* (27).

The GC-MS analysis revealed that 42, 41, and 35 volatile flavor compounds were detected in the *P. yezoensis* sauces fermentated by *L. casei, L. fermentum* and the control group, respectively. These volatile compounds were responsible for *P. yezoensis* sauce flavor. Aldehydes are generated by fat oxidation, providing a fat scent (28). In the *L. casei* fermentation group one aldehyde was detected at Day 7, and 8 aldehydes were found at Day 21. The portion of aldehyde in the total volatile components increased from 2.71% at Day 7 to 46.45% at Day 21. However, in the *L. fermentum* group, the relative amount of aldehydes dropped from 25.53 to 6.64% and the number of compounds dropped from 6 to 3. In the control group, the ratio of aldehyde species increased from 2.49 to 8.47%, and the number of aldehyde species increased from 2 to 5. The unsaturated aldehydes have attractive aromas (29). For example, benzaldehyde possesses pleasant fruity flavor, and furfural refers to thew sweet and almond scent. These components can contribute to the enhancement of the sauce flavor (30).

Alcohols are produced by the microbial metabolism including the breakdown of unsaturated fatty acids and the reduction of base chemicals, which impart a clove scent (31). A total of 8 alcoholic compounds were detected from the six groups, including 2,3-Butanediol, [S-(R^*^,R^*^)], 2,3-butanediol, 2-furanmethanol, benzyl alcohol, phenylethyl alcohol and 2,3-Butanediol, [R-(R^*^,R^*^)], etc. The relative content of 2,3-Butanediol, [R-(R^*^,R^*^)] increased up to 40.91% at Day 7 of *L. casei* fermentation, and then decreased to 1.71% at Day 21 of fermentation, thus providing little contribution to the overall flavor.

Hydrocarbon compounds were identified in *P. yezoensis* sauces, including β-ocimene, eicosane, heptadecane, 8-heptadecene, tetradecane and others. Although alkyene compounds were regarded as odorless due to the cleavage of the alkoxygen radical, some olefins generated aldehydes and ketones under specific circumstances still can contribute to the overall flavor of *P. yezoensis* sauces (32). Several alkanes, when degraded under certain circumstances, generated a fishy odor (33). After 21 days of fermentation with *L casei*, the number of hydrocarbon species increased from 4 to 9, with the relative content of 8-heptadecene increased from 7.15 to 19.15%. During fermentation, the relative content of 8-heptadecene in unfermented group increased from 2.21 to 16.48%, but the relative content of 8-heptadecene during *L. fermentum* fermentation was no significant difference. It was widely established that 8-heptadecene tadecene was a distinctive flavor compound found in a wide variety of macroalgae (34).

*P. yezoensis* sauces contained a total of four acid compounds, namely acetic acid, butanoic acid, dodecane, 4-methyl, and 1-non-adecene. Among them, acetic acid is primarily an enzymatic response in the lipogenesis enzyme pathway, which has an oily fishy smell. Also, acetic acid may create ester bonds with sugar to enhance the flavor (35). The *L. casei* fermentation reduced the concentration of acetic acid, consequently atteunating the pungent smell of *P. yezoensis* sauces (36). At the same time, the release of acetic acid indicates that *L. casei* has the ability to metabolize lactic acid (37).

Esters are formed by esterifying carboxylic acid and alcoho, which are usually related with fruit or floral scents, and contributing to the flavor of food products (38, 39). Ketones with a unique fruit smell, can be generated by heat oxidation or breakdown of amino acids (40). Pyrazines are nitrogen-containing heterocyclic compounds that are an intermediate in the Millard reaction (41). They often have a barbeque and nut flavor, an excellent dispersed fragrance, and an extreme deficient concentration. These volatile compounds were detected in *P. yezoensis* sauce fermented by *L. casei*. Therefore, it was determined that fermenting *P. yezoensis* sauce with *L. casei* produces the finest flavor.

## Conclusion

In this study, three LAB strains fermented sandwich seaweed processing waste to prepare *P. yezoensis* sauce. The detection and analysis of the three LAB strains fermented *P. yezoensis* sauces revealed that the *L. casei* was able to adapt to the fermentation environment and had high protease vitality. Sensory evaluation results showed that fermentation of *L. casei* could enhanced the acceptability of *P. yezoensis* sauce. Following GC-MS detection, *P. yezoensis* sauce generated 42 volatile flavor compounds by fermentation of *L. casei*. These volatile chemicals are essential for *P. yezoensis* sauce taste. The antioxidant activity of *P. yezoensis* sauces fermented by *L. casei* was also higher. Overall, *L. casei* fermented *P. yezoensis* sauce had the best flavor and was appropriate for producing *P. yezoensis* sauce meals. The fermented *P. yezoensis* sauces has a distinctive taste and excellent nutritional value for the elderly, children and babies. This study can provide guidance for the development of LAB fermented *P. yezoensis* sauces in future studies.

## Data Availability Statement

The original contributions presented in the study are included in the article/Supplementary Material, further inquiries can be directed to the corresponding author/s.

## Author Contributions

JY performed the experiments, analyzed the data, and wrote the manuscript. TG and FG analyzed the data and wrote the manuscript. HS, ZC, and ZW analyzed and discussed the data. SW, PS, and YT provided samples and discussed the data. WW designed the research content, analyzed the data, and modified the manuscript. All authors have read and agreed to the published version of the manuscript.

## Funding

This study was supported by the Project funded by Opening Foundation of Key Laboratory of high-tech veterinary biopharmaceutical research, Jiangsu province (No. NSFK201803), National Natural Science Foundation of China (Nos. 32100037 and 32172284), Natural Science Research General Project of Jiangsu Higher Education Institutions (No. 20KJB550008), China Postdoctoral Science Foundation (No. 2019M661767), Natural Science Foundation of Jiangsu Province (No. BK20201028), Jiangsu Planned Projects for Postdoctoral Research Funds (No. 2021K316C), Jiangsu Ocean University Research Funds (No. KQ17028), and Priority Academic Program Development of Jiangsu Higher Education Institutions (PAPD).

## Conflict of Interest

The authors declare that the research was conducted in the absence of any commercial or financial relationships that could be construed as a potential conflict of interest.

## Publisher's Note

All claims expressed in this article are solely those of the authors and do not necessarily represent those of their affiliated organizations, or those of the publisher, the editors and the reviewers. Any product that may be evaluated in this article, or claim that may be made by its manufacturer, is not guaranteed or endorsed by the publisher.

## References

[1] Jin C Wang J Wang S Xu X. Porphyra species: a mini-review of its pharmacological and nutritional properties. J Med Food. (2016) 19:111–9. 10.1089/jmf.2015.3426 26653974

[2] Watanabe F Takenaka S Katsura H Miyamoto E Nakano Y. Characterization of a vitamin B12 compound in the edible purple laver. porphyra yezoensis. J Agric Chem Soc Japan. (2000) 64:2712–5. 10.1271/bbb.64.2712 11210144

[3] Venkatraman KL Syed AA Indumathi P Mehta A. Vitpor ai, a coagulation factor xiia inhibitor from *porphyra yezoensis: In vivo* mode of action and assessment of platelet function analysis. Prot Pept Lett. (2020) 27:243–50. 10.2174/0929866526666191026111056 31738131

[4] Qian L Zhou Y Ma JX. Hypolipidemic effect of the polysaccharides from porphyra yezoensis. Int J Biol Macromol. (2014) 68:48–9. 10.1016/j.ijbiomac.2014.04.004 24736124

[5] Tian Y Jiang Y Guo Y Zhao Y Li N Yao L. Research progress on nutritional quality and edible value of *Porphyra*. J Food Saf Qual. (2021) 12:4929–36. 10.19812/j.cnki.jfsq11-5956/ts.2021.12.033

[6] Zhang X Luo J Zhuo D He Y Zhou H Ren D. Fermentation technology of special *Porphyra haitanensis* sauce. J Food Saf Qual. (2020) 11:2609–16. 10.19812/j.cnki.jfsq11-5956/ts.2020.08.047

[7] Fan L Shi X. Technology of fermentative *Porphyra* sauce. Food Sci Technol. (2015) 40:269–72. 10.13684/j.cnki.spkj.2015.03.064

[8] Uchida M Miyoshi T Yoshida G Niwa K Mori M Wakabayashi H. Isolation and characterization of halophilic lactic acid bacteria acting as a starter culture for sauce fermentation of the red alga nori (*porphyra yezoensis*). J Appl Microbiol. (2014) 116:1506–20. 10.1111/jam.12466 24494732

[9] Ryu S. Study on manufactute of porphyran jam and eppiciency extraction method of porphyran from *Porphyra yezoensis*. J Korean Appl Sci Technol. (2013) 30:504–17. 10.12925/jkocs.2013.30.3.504

[10] Yuasa M Shimada A Matsuzaki A Eguchi A Tominaga M. Chemical composition and sensory properties of fermented citrus juice using probiotic lactic acid bacteria. Food Biosci. (2021) 39:100810. 10.1016/j.fbio.2020.100810

[11] Chen Y Wang Y Chen J Tang H Wang C Li Z. Bioprocessing of soybeans (*Glycine max* L.) by solid-state fermentation with *Eurotium cristatum* YL-1 improves total phenolic content, isoflavone aglycones, and antioxidant activity. RSC Adv. (2020) 10:16928–41. 10.1039/C9RA10344A PMC9053166 35496929

[12] Zhang S. Determination of the relationship between protease enzyme activity and number of psychrophilic bacteria in fresh milk in Harbin. China Dairy Ind. (2012) 40:41–9. 10.3969/j.issn.1001-2230.2012.07.012

[13] Apa B Yha B Rui M Khe A Lpt C Mc A. Combination of solid phase microextraction and low energy electron ionization gas chromatography-quadrupole time-of-flight mass spectrometry to meet the challenges of flavour analysis. Talanta. (2021) 235:122793. 10.1016/j.talanta.2021.122793 34517651

[14] Augustini A Sielemann S Telgheder U. Strategy for the identification of flavor compounds in e-liquids by correlating the analysis of GCxIMS and GC-MS. Talanta. (2021) 230:122318. 10.1016/j.talanta.2021.122318 33934782

[15] Yang J Lu J Zhu Q Tao Y Zhu Q Guo C. Isolation and characterization of a novel *Lactobacillus plantarum* MMB-07 from traditional suanyu for *Acanthogobius hasta* fermentation. J Biosci Bioeng. (2021) 132:161–6. 10.1016/j.jbiosc.2020.12.016 33972168

[16] Tang WZ Wang B Wang MM Wang MM. Ultrasound-assisted extraction of *Osmanthus fragrans* fruit oil and evaluation of its fatty acid composition, physicochemical properties and antioxidant activity. J Appl Res Med Aroma. (2021) 25:100331. 10.1016/j.jarmap.2021.100331

[17] Xu J Xu L Zhou Q Hao S Zhou T Xie H. Enhanced in vitro antioxidant activity of polysaccharides from Enteromorpha prolifera by enzymatic degradation. J Food Biochem. (2016) 40:275–83. 10.1111/jfbc.1221828363657

[18] Muhialdin BJ Hussin A K Adum H Hamid AA Jaafar AH. Metabolomic changes and biological activities during the lacto-fermentation of jackfruit juice using *lactobacillus casei* atcc334. LWT-Food Sci Technol. (2021) 141:110940. 10.1016/j.lwt.2021.110940

[19] Wang Y Tao Y Zhang X Shao S Han Y Chu D. Metabolic profile of ginkgo kernel juice fermented with lactic acid bacteria: a potential way to degrade ginkgolic acids and enrich terpene lactones and phenolics. Process Biochem. (2018) 76:25–33. 10.1016/j.procbio.2018.11.006

[20] Li W Wang T. Effect of solid-state fermentation with *Bacillus subtilis* lwo on the proteolysis and the antioxidative properties of chickpeas. Int J Food Microbiol. (2020) 338:108988. 10.1016/j.ijfoodmicro.2020.108988 33267968

[21] Li S Jin Z Hu D Yang W Chen X. Effect of solid-state fermentation with *Lactobacillus casei* on the nutritional value, isoflavones, phenolic acids and antioxidant activity of whole soybean flour. LWT Food Sci Technol. (2020) 125:109264. 10.1016/j.lwt.2020.109264

[22] Song J Bi J Chen Q. Assessment of sugar content, fatty acids, free amino acids, and volatile profiles in jujube fruits at different ripening stages. Food Chem. (2019) 270:344–52. 10.1016/j.foodchem.2018.07.102 30174057

[23] Isaka S Cho K Nakazono S Abu R Ueno M Kim D. Antioxidant and anti-inflammatory activities of porphyran isolated from discolored nori (*porphyra yezoensis*). Int J Biol Macromol. (2015) 74:68–75. 10.1016/j.ijbiomac.2014.11.043 25499893

[24] Wang F Kong LM Xie YY Wang C Zhou T. Purification, structural characterization, and biological activities of degraded polysaccharides from *Porphyra yezoensis*. J Food Biochem. (2021) 45:e13661. 10.1111/jfbc.13661 33595138

[25] Li S Tao Y Li D Wen G Zhou J Manickam S. Fermentation of blueberry juices using autochthonous lactic acid bacteria isolated from fruit environment: fermentation characteristics and evolution of phenolic profiles. Chemosphere. (2021) 276:130090. 10.1016/j.chemosphere.2021.130090 33740651

[26] Cho TJ Min SR. Health functionality and quality control of laver (*Porphyra, Pyropia*): current issues and future perspectives as an edible seaweed. Marine Drugs. (2020) 18:14. 10.3390/md18010014 31877971 PMC7024182

[27] Zubaidah E Arum MS Widyaningsih TD Rahayu AP. Sauerkraut with the addition of *lactobacillus casei*: effects of salt and sugar concentrations on fermentation and antioxidant activity. Curr Nutr Food Sci. (2020) 16:1265–9. 10.2174/1573401316666200217112642

[28] Widjaja R Craske JD Wootton M. Comparative studies on volatile components of non-fragrant and fragrant rices. J Sci Food Agric. (2015) 70:151–61. 10.1002/(SICI)1097-0010(199602)70:2<151::AID-JSFA478>3.0.CO;2-U

[29] Jiye A Trygg J Gullberg J Johansson AI Jonsson P Antti H. Extraction and GC/MS analysis of the human blood plasma metabolome. Anal Chem. (2005) 77:8086–94. 10.1021/ac051211v 16351159

[30] Cao R Hu M Zhao L Wang L Liu Q. Flavor characteristics of different crops of laver (*Porphyra yezoensis*) during one harvest cycle. J Ocean U China. (2021) 20:213–20. 10.1007/s11802-021-4447-3

[31] Lee SM Seo BC Kim YS. Volatile compounds in fermented and acid-hydrolyzed soy sauces. J Food Sci. (2006) 71:C146–56. 10.1111/j.1365-2621.2006.tb15610.x

[32] Giannetti V Mariani MB Mannino P. Volatile fraction analysis by HS-SPME/GC-MS and hemometric modeling for traceability of apples cultivated in the Northeast Italy. Food Control. (2017) 78:215–21. 10.1016/j.foodcont.2017.02.036

[33] Chakraborty K Joseph D. Production and characterization of refined oils obtained from Indian oil sardine (*Sardinella longiceps*). J Agric Food Chem. (2015) 63:998–1009. 10.1021/jf505127e 25547196

[34] Murathan ZT Zarifikhosroshahi M Kafkas NE. Determination of fatty acids and volatile compounds in fruits of rosehip (*Rosa L*.) species by HS-SPME/GC-MS and Im-SPME/GC-MS techniques. Turk J Agric For. (2016) 40:269–79. 10.3906/tar-1506-50

[35] Lalel H Singh Z Tan SC. Glycosidically-bound aroma volatile compounds in the skin and pulp of 'kensington pride' mango fruit at different stages of maturity. Postharvest Biol Tec. (2003) 29:205–18. 10.1016/S0925-5214(02)00250-8

[36] Nishino N Touno E. Ensiling characteristics and aerobic stability of direct-cut and wilted grass silages inoculated with *Lactobacillus casei* or *Lactobacillus buchneri*. J Sci Food Agric. (2005) 85:1882–8. 10.1002/jsfa.218925855820

[37] Li S Chen C Ji Y Lin J Chen X Qi B. Improvement of nutritional value, bioactivity and volatile constituents of quinoa seeds by fermentation with *lactobacillus casei*. J Cereal Sci. (2018) 84:83–9. 10.1016/j.jcs.2018.10.008

[38] Jabalpurwala F Gurbuz O Rouseff R. Analysis of grapefruit sulphur volatiles using SPME and pulsed flame photometric detection. Food Chem. (2010) 120:296–303. 10.1016/j.foodchem.2009.09.079

[39] Xiao Y Huang Y Chen Y Fan Z Chen R He C. Effects of solid-state fermentation with *Eurotium cristatum* YL-1 on the nutritional value, total phenolics, isoflavones, antioxidant activity, and volatile organic compounds of black soybeans. Agronomy. (2021) 11:1029. 10.3390/agronomy11061029

[40] Sarnoski PJ O'Keefe SF Jahncke ML Mallikarjunan P Flick GJ. Analysis of crab meat volatiles as possible spoilage indicators for blue crab (*Callinectes sapidus*) meat by gas chromatography-mass spectrometry. Food Chem. (2017) 122:930–5. 10.1016/j.foodchem.2010.03.069

[41] Pini GF Brito E García NHP Valente A Augusto F. A headspace solid phase microextraction (HS-SPME) method for the chromatographic determination of alkylpyrazines in cocoa samples. J Brazil Chem Soc. (2004) 15:267–71. 10.1590/S0103-50532004000200017

