# Supplementary material for: Porphyra yezoensis Sauces Fermented With Lactic Acid Bacteria: Fermentation Properties, Flavor Profile, and Evaluation of Antioxidant Capacity in vitro
Source: Front Nutr. 2022 Jan 13;8:810460. doi: 10.3389/fnut.2021.810460 (PMC8805458; doi:10.3389/fnut.2021.810460)
Supplement: Supplementary file 1 [file Table_1.docx]

**SUPPLEMENTARY MATERIALS**

***Porphyra yezoensis* Sauces Fermented with Lactic Acid Bacteria: Fermentation Properties, Flavor Profile, and Evaluation of Antioxidant Capacity *in vitro***

Jie Yang^1, 2, 3^, Tengqi Gao^1^, Feng Ge^1^, Hao Sun^1^, Zihang Cui ^1^, Zhen Wei^1, 3^, Shujun Wang^1, 3^, Pau Loke Show^4^, Yang Tao^5^, Wenbin Wang^1, 3*^

^1^Jiangsu Key Laboratory of Marine Bioresources and Environment/Jiangsu Key Laboratory of Marine Biotechnology, Jiangsu Ocean University, Lianyungang, 222005, China

^2^Jiangsu Key Laboratory of High-Tech Research and Development of Veterinary Biopharmaceuticals, Jiangsu Agri-animal Husbandry Vocational College, Taizhou, 225300, China

^3^Co-Innovation Center of Jiangsu Marine Bio-industry Technology, Jiangsu Ocean University, Lianyungang 222005, China

^4^Department of Chemical and Environmental Engineering, Faculty of Science and Engineering, University of Nottingham Malaysia, Jalan Broga, 43500 Semenyih, Selangor Darul Ehsan, Malaysia.

^5^College of Food Science and Technology, Nanjing Agricultural University, 1 Weigang, Nanjing 210095, China

*Corresponding authors:

Wenbin Wang, Email: wenbin66@jou.edu.cn

Jiangsu Key Laboratory of Marine Bioresources and Environment, Jiangsu Ocean University, Lianyungang 222005, China.

**Table S1.** Volatile compounds identified of *P. yezoensis* sauce fermented with *L. fermentum* and *L. casei*

| **Compound Name** | Control-7 days | | *L. fermentum*-7 days | | *L. casei*-7 days | | Control-21 days | | *L. fermentum*-21 days | | *L. casei*-21 days | |
| --- | --- | --- | --- | --- | --- | --- | --- | --- | --- | --- | --- | --- |
|  | Content (mg/L) | Relative content (%) | Content (mg/L) | Relative content (%) | Content (mg/L) | Relative content (%) | Content (mg/L) | Relative content (%) | Content (mg/L) | Relative content (%) | Content (mg/L) | Relative content (%) |
| **ketones** |  |  |  |  |  |  |  |  |  |  |  |  |
| trans-β-Ionone | ND | ND | 7.36 | 0.59 | 10.97 | 0.23 | ND | ND | 11.65 | 0.45 | ND | ND |
| 3-Buten-2-one, 4-(2,6,6-trimethyl-1-cyclohexen-1-yl)- | ND | ND | ND | ND | ND | ND | 16.53 | 0.37 | ND | ND | 28.52 | 0.9 |
| **aldehydes** |  |  |  |  |  |  |  |  |  |  |  |  |
| Nonanal | 36.76 | 0.71 | 42.26 | 3.39 | ND | ND | ND | ND | ND | ND | ND | ND |
| Benzaldehyde | 91.99 | 1.78 | 108.72 | 8.72 | 128.90 | 2.71 | 115.91 | 2.62 | 116.73 | 4.52 | 374.26 | 11.79 |
| 2-Pentenal, 2-methyl- | ND | ND | 54.29 | 4.35 | ND | ND | ND | ND | ND | ND | ND | ND |
| Furfural | ND | ND | 99.86 | 8.01 | ND | ND | 109.98 | 2.49 | ND | ND | 523.75 | 16.49 |
| 2-Furancarboxaldehyde, 5-methyl- | ND | ND | 6.01 | 0.48 | ND | ND | ND | ND | ND | ND | 52.78 | 1.66 |
| 1-Cyclohexene-1-carboxaldehyde, 2,6,6-trimethyl- | ND | ND | ND | ND | ND | ND | ND | ND | ND | ND | 19.65 | 0.62 |
| 5-Hydroxymethylfurfural | ND | ND | 7.24 | 0.58 | ND | ND | ND | ND | ND | ND | ND | ND |
| 2-Octenal, (E)- | ND | ND | ND | ND | ND | ND | 61.76 | 1.4 | ND | ND | ND | ND |
| 2,4-Decadienal, (E,E)- | ND | ND | ND | ND | ND | ND | 32.98 | 0.75 | ND | ND | 43.12 | 1.36 |
| Benzaldehyde, 3,5-dimethyl- | ND | ND | ND | ND | ND | ND | 53.29 | 1.21 | 32.29 | 1.25 | ND | ND |
| 2,4-Heptadienal, (E,E)- | ND | ND | ND | ND | ND | ND | ND | ND | 22.36 | 0.87 | ND | ND |
| Benzaldehyde, 3-ethyl- | ND | ND | ND | ND | ND | ND | ND | ND | ND | ND | 15.66 | 0.49 |
| Benzaldehyde, 3,4-dimethyl- | ND | ND | ND | ND | ND | ND | ND | ND | ND | ND | 15.04 | 0.47 |
| Benzaldehyde, 2,4-dimethyl- | ND | ND | ND | ND | ND | ND | ND | ND | ND | ND | 431.02 | 13.57 |
| **acids** |  |  |  |  |  |  |  |  |  |  |  |  |
| Acetic acid | 203.43 | 3.93 | 68.67 | 5.51 | 484.76 | 10.17 | 324.57 | 7.34 | 304.73 | 11.81 | 240.17 | 7.56 |
| Butanoic acid | 47.88 | 0.92 | ND | ND | ND | ND | ND | ND | ND | ND | ND | ND |
| Dodecane, 4-methyl- | ND | ND | ND | ND | ND | ND | ND | ND | ND | ND | 20.83 | 0.66 |
| 1-Nonadecene | ND | ND | ND | ND | ND | ND | ND | ND | 5.43 | 0.21 | ND | ND |
| **esters** |  |  |  |  |  |  |  |  |  |  |  |  |
| Hexadecanoic acid, ethyl ester | 2.18 | 0.04 | ND | ND | ND | ND | ND | ND | ND | ND | ND | ND |
| Butyrolactone | ND | ND | 7.02 | 0.56 | ND | ND | ND | ND | ND | ND | ND | ND |
| Formic acid, octyl ester | ND | ND | ND | ND | ND | ND | 23 | 0.52 | ND | ND | ND | ND |
| 2(4H)-Benzofuranone, 5,6,7,7a-tetrahydro-4,4,7a-trimethyl- | ND | ND | ND | ND | ND | ND | ND | ND | ND | ND | 14.62 | 0.46 |
| **alcohols** |  |  |  |  |  |  |  |  |  |  |  |  |
| 2,3-Butanediol, [S-(R*,R*)]- | 1508.68 | 29.13 | ND | ND | ND | ND | ND | ND | ND | ND | ND | ND |
| 2,3-Butanediol | 1727.56 | 33.36 | 49.65 | 3.98 | ND | ND | ND | ND | ND | ND | ND | ND |
| 2-Furanmethanol | 56.93 | 1.10 | 236 | 18.92 | 280.87 | 5.89 | 20.9 | 0.47 | 179.4 | 6.95 | 128.05 | 4.03 |
| Benzyl alcohol | 14.82 | 0.29 | ND | ND | 27.53 | 0.58 | 30.16 | 0.68 | 15.77 | 0.61 | 29.87 | 0.94 |
| Phenylethyl Alcohol | 89.69 | 1.73 | 14.69 | 1.18 | 79.03 | 1.66 | 66.29 | 1.5 | 43.22 | 1.67 | 45.38 | 1.43 |
| 1-Hexanol | ND | ND | ND | ND | 47.89 | 1.01 | ND | ND | ND | ND | ND | ND |
| 2,3-Butanediol, [R-(R*,R*)]- | ND | ND | 44.9 | 3.6 | 1949.38 | 40.91 | 1917.24 | 43.36 | 165.48 | 6.41 | 54.23 | 1.71 |
| 1-Octanol | ND | ND | ND | ND | ND | ND | ND | ND | 19.67 | 0.76 | 25.47 | 0.8 |
| **phenols** |  |  |  |  |  |  |  |  |  |  |  |  |
| Phenol, 2-methoxy- | 268.93 | 5.19 | 6.92 | 0.55 | 245.8 | 5.16 | 118.99 | 2.69 | 482.33 | 18.69 | 36.36 | 1.14 |
| Phenol | 92.51 | 1.79 | ND | ND | 23.6 | 0.5 | 42.7 | 0.97 | 13.12 | 0.51 | ND | ND |
| 2-Methoxy-4-vinylphenol | 46.71 | 0.90 | 8.84 | 0.71 | 46.59 | 0.98 | ND | ND | 54.54 | 2.11 | ND | ND |
| 1,3-Benzodioxol-5-ol | ND | ND | 16.42 | 1.32 | ND | ND | ND | ND | ND | ND | ND | ND |
| **hydrocarbons** |  |  |  |  |  |  |  |  |  |  |  |  |
| β-Ocimene | 37.18 | 0.72 | ND | ND | 39.44 | 0.83 | ND | ND | 28.67 | 1.11 | 43.05 | 1.36 |
| Eicosane | ND | ND | 4.71 | 0.38 | ND | ND | ND | ND | 25.89 | 1 | 6.73 | 0.21 |
| Heptadecane | 17.40 | 0.34 | 25.4 | 2.04 | 50.64 | 1.06 | 94.6 | 2.14 | 49.44 | 1.92 | 88.18 | 2.78 |
| 8-Heptadecene | 114.68 | 2.21 | 157.13 | 12.6 | 340.86 | 7.15 | 728.9 | 16.48 | 312.69 | 12.12 | 608.14 | 19.15 |
| Dodecane | ND | ND | 8.1 | 0.65 | ND | ND | ND | ND | ND | ND | ND | ND |
| Tetradecane | ND | ND | 10.79 | 0.87 | 25.7 | 0.54 | 65.23 | 1.48 | 24.93 | 0.97 | ND | ND |
| Pentadecane | ND | ND | ND | ND | ND | ND | 34.33 | 0.78 | ND | ND | 49.38 | 1.56 |
| Pentacosane | ND | ND | ND | ND | ND | ND | ND | ND | 28.48 | 1.1 | ND | ND |
| Heptacosane | ND | ND | ND | ND | ND | ND | ND | ND | ND | ND | 27.15 | 0.86 |
| Hexadecane | ND | ND | ND | ND | ND | ND | ND | ND | ND | ND | 8.94 | 0.28 |
| 1,3-Cyclooctadiene | ND | ND | ND | ND | ND | ND | ND | ND | ND | ND | 23.97 | 0.75 |
| 3-Heptadecene, (Z)- | ND | ND | 5.64 | 0.45 | ND | ND | ND | ND | ND | ND | ND | ND |
| Naphthalene | ND | ND | ND | ND | ND | ND | ND | ND | ND | ND | 10.69 | 0.34 |
| **pyrazines** |  |  |  |  |  |  |  |  |  |  |  |  |
| Pyrazine, methyl- | ND | ND | 7.41 | 0.59 | ND | ND | ND | ND | ND | ND | 25.33 | 0.8 |
| Pyrazine, 3-ethyl-2,5-dimethyl- | ND | ND | 34.45 | 2.76 | 78.41 | 1.65 | 74.7 | 1.69 | 42.18 | 1.63 | 54.68 | 1.72 |
| Pyrazine, tetramethyl- | 161.26 | 3.11 | ND | ND | ND | ND | ND | ND | ND | ND | ND | ND |
| Pyrazine, 2-ethyl-5-methyl- | ND | ND | ND | ND | ND | ND | 23.13 | 0.52 | ND | ND | 15.43 | 0.49 |
| Pyrazine, 3,5-diethyl-2-methyl- | ND | ND | ND | ND | ND | ND | ND | ND | ND | ND | 15.09 | 0.48 |
| Pyrazine, 3,5-dimethyl-2-propyl- | ND | ND | 7.17 | 0.57 | ND | ND | 17.14 | 0.39 | ND | ND | ND | ND |
| **furans** |  |  |  |  |  |  |  |  |  |  |  |  |
| Ethanone, 1-(2-furanyl)- | 37.09 | 0.72 | 25.66 | 2.06 | 70.78 | 1.49 | 74.14 | 1.68 | 56.53 | 2.19 | 52.83 | 1.66 |
| 2,2'-Bifuran | ND | ND | ND | ND | ND | ND | 286.37 | 6.48 | ND | ND | 12.58 | 0.4 |
| **benzodiazepines** |  |  |  |  |  |  |  |  |  |  |  |  |
| Benzene, 1,3-dimethyl- | ND | ND | 8.68 | 0.7 | ND | ND | ND | ND | ND | ND | ND | ND |
| **others** |  |  |  |  |  |  |  |  |  |  |  |  |
| 1,3,6-Octatriene, 3,7-dimethyl-, (Z)- | ND | ND | 35.3 | 2.83 | ND | ND | 32.92 | 0.74 | ND | ND | ND | ND |
| Dimethyl trisulfide | ND | ND | 5.83 | 0.47 | ND | ND | ND | ND | ND | ND | 12.12 | 0.38 |
| Ethanone, 1-(1H-pyrrol-2-yl)- | 7.47 | 0.14 | 4.2 | 0.34 | ND | ND | ND | ND | ND | ND | ND | ND |
| Benzonitrile | ND | ND | ND | ND | ND | ND | 55.39 | 1.25 | ND | ND | ND | ND |
| Bicyclo[3.1.1]hept-2-ene, 2,6-dimethyl-6-(4-methyl-3-pentenyl)- | ND | ND | 9.26 | 0.74 | ND | ND | ND | ND | ND | ND | ND | ND |
| Heptadecane, 4-methyl- | ND | ND | ND | ND | ND | ND | ND | ND | ND | ND | 13.77 | 0.43 |
| 3-Buten-2-one, 4-(2,6,6-trimethyl-2-cyclohexen-1-yl)- | ND | ND | ND | ND | ND | ND | ND | ND | ND | ND | 8.6 | 0.27 |
| Oxime-, methoxy-phenyl-_ | 615.13 | 11.88 | 114.9 | 9.21 | 833.82 | 17.5 | ND | ND | 545.31 | 21.13 | ND | ND |
| 2,4,6-Octatriene, 2,6-dimethyl-, (E,Z)- | ND | ND | 3.82 | 0.31 | ND | ND | ND | ND | ND | ND | ND | ND |
